# Static Wettability of Differently Mechanically Treated and Amphiphobic-Coated Aluminium Surfaces

**DOI:** 10.3390/ma13102240

**Published:** 2020-05-13

**Authors:** Nataliia Fedorova, Bettina Ottinger, Vojislav Jovicic, Ana Zbogar-Rasic, Antonio Delgado, Sannakaisa Virtanen

**Affiliations:** 1 Institute of Fluid Mechanics (LSTM), Friedrich-Alexander University (FAU), 91058 Erlangen, Germany; bettina.studium@franken-online.de (B.O.); ana.zbogar-rasic@fau.de (A.Z.-R.); antonio.delgado@fau.de (A.D.); 2 Erlangen Graduate School in Advanced Optical Technologies (SAOT), 91052 Erlangen, Germany; 3 Institute for Surface Science and Corrosion (WW4), Friedrich-Alexander University (FAU), 91058 Erlangen, Germany; virtanen@ww.uni-erlangen.de

**Keywords:** wettability, amphiphobic, superhydrophobic, surface treatment, roughness, contact angle

## Abstract

Wettability, roughness and surface treatment methods are essential for the majority of practical applications, where liquid–solid surface interactions take place. The present study experimentally investigated the influence of different mechanical surface treatment methods on the static wettability of uncoated and amphiphobic-coated aluminium alloy (AlMg_3_) samples, specially focusing on the interaction between surface finishing and coating. Five different surfaces were prepared: as-received substrate, polished, sandpapered, fleece-abraded and sandblasted. After characterisation, the samples were spray-coated using an amphiphobic coating. The characterisation of the uncoated and coated samples involved measurements of the roughness parameters and the apparent contact angles of demineralized water and rapeseed oil. The coating was initially characterised regarding its adhesion to the sample and elevated temperature stability. The applied surface treatments resulted in the scattered sample roughness in the range of Sa = 0.3–15.8 µm, water contact angles of $\theta_{ap,w}$ = 78°–106° and extremely low oil contact angles. Coating the samples more than doubled the surface roughness to Sa = 13.3–29 µm, whereas the initial surface treatment properties (structure, anisotropy, etc.) were entirely repressed by the coating properties. Coating led the water contact angles to increase to $\theta_{ap,w\_coated}$ = 162°–173° and even more pronounced oil contact angles to increase to $\theta_{ap,o\_coated}$ = 139°–150°, classifying the surfaces as superhydrophobic and oleophobic.

## 1. Introduction

Wettability is a physical parameter that describes the affinity of a liquid toward a solid phase. The basics on wetting were introduced by Young [1], Wenzel [2] and Cassie and Baxter [3] more than two hundred years ago. Interest in wettability research was renewed in the 1990s resulting in a tenfold increase in the number of publications during the next ten years [4]. Since approximately 2005, a new wave of research activity on wettability in connection with superhydrophilic and superhydrophobic surfaces has been observed, as the progress in material science, inspired by nature, enabled this type of coatings and surface structuring [4].

Motivation behind this study is related to the recent reports on superhydrophobic surfaces promoting the jumping-droplet condensation process which exhibits high heat transfer coefficients [5,6]. This behaviour could extend the possibilities of different condensation systems for the recovery of the latent heat of the water vapour in the flue gas [7]. Apart from that, further applications and processes dependent on the wettability phenomenon include printing, adhesion, coating, lubrication, air conditioning, desalination, corrosion prevention, oil–water separation, anti-icing, food packaging, etc. [8,9,10,11,12,13,14,15].

In general, static wettability is quantified by the contact angle (θ) which represents the angle between the tangent to the liquid–vapour interface and the solid surface at the three-phase contact line [16]. According to its contact angle, in case of a water droplet, the surface can be [4]:

- superhydrophilic (θ < 10°);
- hydrophilic (θ < 90°);
- hydrophobic (θ > 90°);
- superhydrophobic (θ > 150°).

The prefix “hydro-” is changed to “oleo-” in the case of an oil droplet and to “amphi” when the contact angles of both water and oil are measured [17,18]. Due to the potential for numerous industrial applications, there is wide interest in amphiphobic and superamphiphobic coatings which can repel various types of oil and other low surface tension liquids in addition to water [19].

One of the crucial parameters that influences the contact angle, and therefore wettability of a surface, is its roughness. Many theoretical and experimental studies were performed on this matter for different liquids (water, silicone oil, ethylene glycol, liquid mercury, etc.), placed on a wide range of substrate materials (aluminium, stainless steel, titanium, silica, alumina, polymethylmethacrylate, etc.) [9,20,21,22]. When roughening a surface, its wettability usually decreases, in case of both wetting and non-wetting liquids [22]. Nevertheless, there are exceptions to this generalisation; therefore, each combination of a liquid on a textured surface has to be tested separately.

The surface roughness is influenced by the surface treatment method, since the wetting behaviour is dependent on the orientation and the texture of roughness [21]. There are numerous choices of mechanical, chemical and electrical methods that are commonly used for surface treatments [23]. The wetting properties on differently treated aluminium surfaces were studied, for example, by Torrisi and Scolaro [24], who applied polishing, sandblasting, chemical etching, laser ablation, ion implantation and deposition of metallic nanoparticles on the surface. The experiments demonstrated the low wetting ability in the case of mirror polishing and ion sputtering by water (hydrophobic surfaces), with the contact angles in the range of 94° to 100°. The research done by Kubiak et al. [9] investigated the influence of material properties on the wetting properties by water. The material choice included aluminium alloy, iron alloy, copper, ceramic, plastic (poly-methylmethacrylate) and titanium alloy. The abrasive polishing by different grit sandpapers (80, 400, 600 and 2500) produced mono-directional, morphologically oriented surfaces, with a wide range of surface roughness Ra = 0.15–7.44 µm. The lowest wettability was measured for the aluminium alloy, with the average contact angle of around 83°. Moreover, a similar influence of roughness on the apparent contact angle was found for all the tested materials: the contact angle value dropped in the intermediate roughness range.

The present experimental study was conducted in order to investigate the influence of different mechanical surface treatment methods on the static wettability of uncoated Al alloy surfaces and surfaces coated with a fluoroacrylic solution, containing fluorinated nanoparticles. The following surface treatment methods were applied: polishing, abrasion with fleece, sandpapering and sandblasting. These methods are commonly used for surface finishing and are mostly available in mechanical workshops. They enabled the production of test samples with directionally oriented and direction-independent morphologies within a wide range of areal surface roughness (Sa = 0.3–15.8 µm). The selected roughness parameters were measured for the characterisation of the surface topography, while the static wettability was characterised by the apparent contact angle measurements. Demineralized water and rapeseed oil were chosen as test liquids, representing a higher and a lower surface tension liquids.

Additionally, the applied coating was initially characterised in terms of its temperature stability and adhesion. These characteristics are essential for sustaining a long-term use in industrial applications.

## 2. Materials and Methods 

In this study, an aluminium alloy was chosen as a substrate material for all considered surface treatment methods. It has high thermal conductivity and is encountered as a heat transfer surface in a variety of systems (e.g., heat exchangers, aircrafts, food packaging, electronics).

The substrates—discs of 59 mm × 3 mm in size—were produced by laser cutting from a rolled metal sheet out of AlMg_3_ alloy (3.0% Mg, 0.4% Si, 0.4% Fe). A marking line (3 mm long) was added in the rolling direction at the edge of each sample. It was used for positioning the sample, in the case of the orientation-dependent surface treatment methods, and for tracking the position of the measurement points on the sample.

The present study considered five surface topographies, aiming to obtain different roughness degrees. The samples were named according to their treatment processes: as-received substrate, polished, sandpapered, fleece-abraded and sandblasted. The substrates were untreated after laser-cutting from the rolled aluminium sheet and served as an initial surface for all the treatment methods. A set of four samples was prepared per each method and was afterwards characterised according to the following procedures.

### 2.1. Preparation of Uncoated Samples

With the aim of reducing the surface roughness and producing a set of polished samples, the substrates were sanded by a grinding machine which was operated at approximately 150 min^−1^, using the SiC sandpapers of 320, 500, 800 and 1200 grit. Subsequently, the samples were polished using the respective polishing discs and diamond suspensions with the diamond sizes of 6 µm for 5 min, 3 µm for 4 min and 1 µm for 3 min. Between the individual grinding and polishing steps, the samples were cleaned with acetone in an ultrasonic bath.

Finishing the surface with the sandpaper, abrasive fleece and sandblast aimed to roughen the substrates. Thus, another set of samples (“sandpapered”) was prepared using the 80 grit sandpaper by a belt sander with a rotational speed of 320 min^−1^. The sandpaper was applied in parallel to the rolling direction of the substrate until the relatively uniform structure of the surface was achieved.

Fleece-abraded samples were produced using the abrasive fleece with a fine A280 grit. It modified the substrates in the rolling direction using a satinising machine at approximately 2000 min^−1^, until a uniformly brushed surface structure was visible.

The sandblasted samples were produced by bombarding the substrates with abrasive particles of quartz sand, with a grain diameter of ~200 µm. These were mixed with air and shot onto the surface for 30 s, at the inlet pressure of around 2 bar. The orthogonally oriented substrates were positioned at a distance of 10 cm to the sandblasting nozzle of 4 mm in diameter.

Four samples were produced per each set, corresponding to each mechanical treatment method. The prepared samples were cleaned before further characterization and coating. Each sample was placed in an ultrasonic bath for 1 min, then rinsed with acetone and placed into another ultrasonic bath with ethanol for 1 min. Finally, a sample was rinsed with ethanol and dried under a dryer.

The applied surface treatment methods are mechanical in nature which makes them valuable for practical conditions and mass production. They are relatively simple and available in most mechanical workshops.

### 2.2. Preparation of Coated Samples

Two samples from each set were coated with the amphiphobic coating. Similar coatings are available on the market. The coating consisted of fluoroacrylic solution with fluorinated nanoparticles with the following ingredients: ethyl nonafluoroisobutyl ether (CAS No.: 163702-06-5), ethyl nonafluorobutyl ether (CAS No.: 163702-05-4) and fluoropolymer. The solution was milky white and non-transparent. 

The spray coating was performed using an airbrush gun with a nozzle of 0.4 mm in diameter. The coating liquid was well shaken before the coating process in order to stabilize the sedimented nanoparticles in the suspension. The container of the airbrush gun was filled with 2.5 mL of the coating for each sample. The spraying was carried out at 2 bar pressure, evenly passing the sample from top to bottom and then from left to right. The samples were dried with the coated surface facing upwards for 24 h at room temperature in an ambient air environment.

### 2.3. Measurements

The characterisation of the prepared uncoated and coated samples involved the measurements of the roughness parameters (Sa, Sq, Sz, Sp, Sv, RSm, defined in Table 1), the contact angles to water and the contact angles to oil. The temperature influence and the coating adhesion to the sample were additionally investigated.

#### 2.3.1. Roughness

The surface roughness parameters were measured by means of a confocal laser scanning microscope with a measurement accuracy of 0.2 μm. It had a mounted 405 nm semiconductor laser, and the profile filter was adjusted to λc = 800 µm for all measurements. 

**Figure 1:**
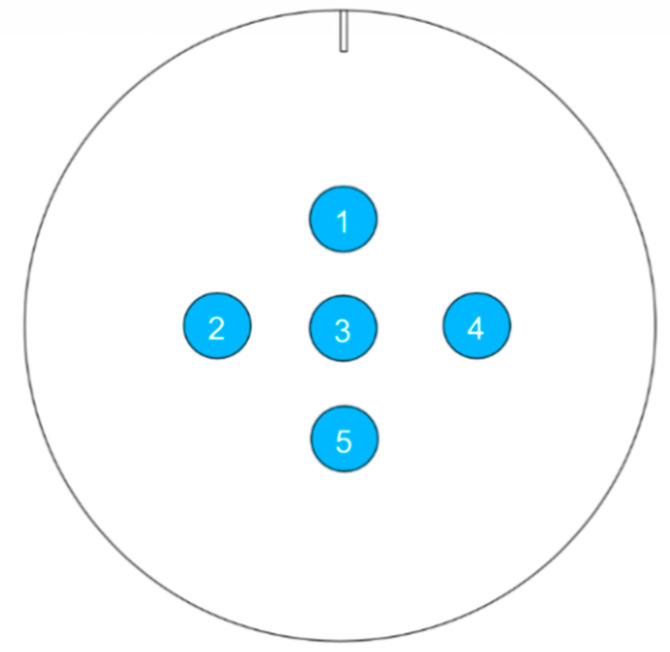
Schematic of the five measurement points on the sample, depending on the rolling direction (indicated by a line) for the roughness determination.

In order to analyse the largest possible area of the sample, a 5× objective lens was used. In this way, each measurement point occupied an area of 6.55 mm^2^ (2560 µm × 2560 µm). In total five points were measured per sample (Figure 1), arranged closer to the centre to avoid defects which can be found more often at the edges. The measurement points were located at a distance of approximately 15 mm from the middle point (Point 3). Thus, these five points per sample were used to obtain an average value of each roughness parameter.

The roughness parameters, selected for the characterisation of the prepared samples, are summarized in Table 1. The arithmetic mean height (Sa), root mean squared height (Sq), maximum height (Sz), maximum peak height (Sp) and maximum valley depth (Sv) are areal roughness parameters according to [25]. The mean width of the profile elements (RSm) is a linear roughness parameter according to [26].

#### 2.3.2. Contact Angle

The sessile drop contact angles of the test liquids on the uncoated and coated aluminium samples were measured by the Drop Shape Analyser (KRÜSS DSA100, Krüss GmbH, Hamburg, Germany) which had the measurement accuracy of 0.3°. The device consisted of the camera and the lens, the sample positioning system, the monochromatic LED light source and the software-controlled dosing system.

Two liquids were tested: demineralized water and rapeseed oil. The influence of the droplet volume on the contact angle was reported to be low, in the range 1–10 µL [29]. Therefore, a droplet of (3 ± 1) µL in volume was freely placed on the sample for 45 s at room conditions. During this time, the apparent contact angle was recorded and measured using the software (DSA4, Krüss GmbH, Hamburg, Germany) and the Young–Laplace model. The applied Young–Laplace model was suitable for our conditions, since on all the samples, the measured contact angles were higher than 10°, the droplet was statically deposited on the surface and its contour shape was quite symmetrical from the left-hand and right-hand sides. The recordings in time helped to take into account the change of the base diameter by the liquid spreading on the rough surfaces. In addition to the contact angle, further parameters, such as the base diameter and the droplet volume, were determined for each measurement.

**Figure 2:**
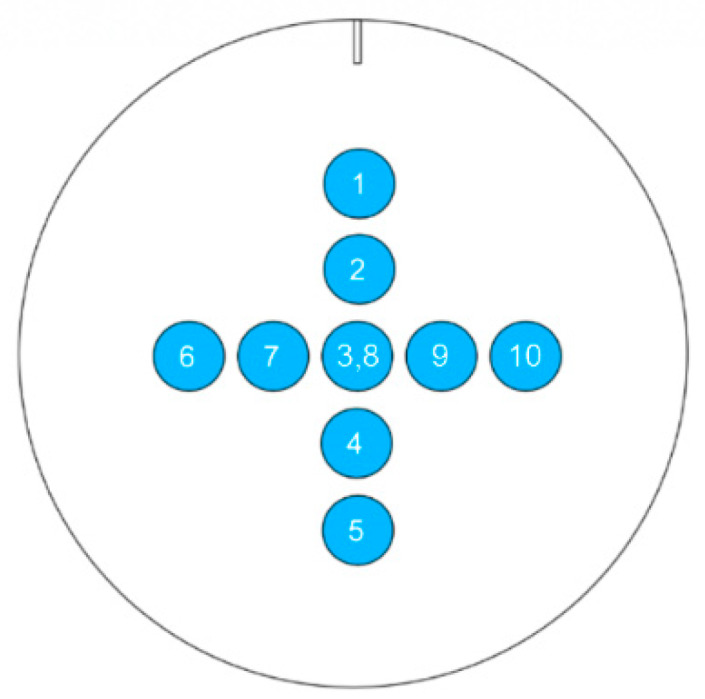
Schematic of the ten measurement points on the sample, depending on the rolling direction (indicated by a line) for the contact angle determination.

In total, ten droplets (one after another) were measured for each sample, which were positioned as schematically presented in Figure 2, at a distance of approximately 10 mm from the middle point (Point 3 and 8) and from each other. The results were averaged over 45 s for droplets 1–10, to obtain the apparent contact angle ($\theta_{ap}$), and separately averaged for droplets 1–5 ($\theta_{ap\_1-5}$) and 6–10 ($\theta_{ap\_6-10}$), to investigate the influence of the directionally oriented roughness. Furthermore, the ratio between the base diameters of the third to the eighth droplet (BD3/BD8) was determined, since the measurement points were purposefully positioned in the same area.

**Figure 3:**
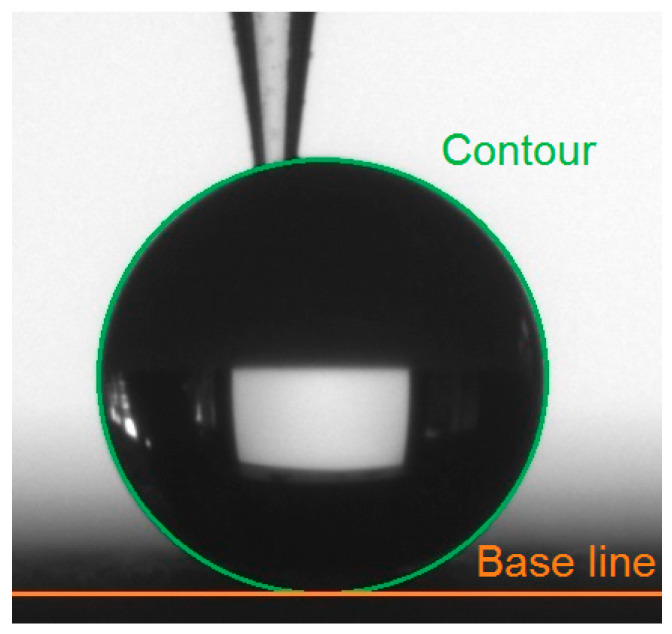
A water droplet on a coated sample with an inserted glass needle tip.

When measuring the water contact angles on the coated samples, it was impossible to freely put the water droplet on the horizontal surface, since it immediately rolled off from the strongly repellent coating. Therefore, the measurement method was modified for this case. The water droplet of 2 µL in volume was formed on the needle, brought in the direct contact with the coated sample and the rest 1 µL was added. As Figure 3 shows, the glass needle tip was inserted into the droplet until no distortion of the drop contour was observed and the contact angle was measured. The applied Young–Laplace model neglected the small area where the needle entered the droplet.

#### 2.3.3. Coating Characterization 

The temperature stability of the coating was verified by heating the coated substrates in a drying oven. One coated substrate was kept three times inside the oven for one hour at 50 °C. Between the heating stages, the samples were cooled for 1 h at room conditions, and the water contact angles were determined at the end of each cooling. Another coated substrate stayed at 50 °C for 10 min, afterwards it was cooled for 1 h and the water and oil contact angles were measured.

The adhesion of the coating towards the substrate was tested by an initial fast adhesion test, named tape test, according to the FINAT method FTM 21, which is used for the examination of the adhesion of paints and varnishes [30]. The stronger adhesive tape, named “tesafilm^®^ kristall-klar” (57315), was attached to the left side of the sample, while the weaker “tesa^®^ Malerband Tapeten” (56260) was applied to the right side of the sample. The middle part of the sample was kept untouched and served as a reference. Each tape stripe was slightly pressed to the sample, following the rolling direction marked with the dash. Afterwards, the tape was removed with a constant speed from the surface, at an angle of 180°. The removal of the coating was assessed visually as following: if the coating is completely removed, the adhesion is poor; if only small particles are removed, the adhesion is very good [30,31]. The water contact angle was afterwards measured by placing 4 droplets in a row on each of the three parts of the sample.

#### 2.3.4. Averaging and Standard Deviations

The deviation of the measured value from the average value was assessed by the multiple repetition of each measurement. For this purpose, the values of each parameter, measured per sample, were averaged and the standard deviations were calculated, according to the following Equations:

(7) $$\bar{x}=\frac{1}{n}\times \overset{n}{\underset{i=1}{\sum }}x_{i}$$

(8) $$\sigma =\sqrt{\frac{1}{n}\times \overset{n}{\underset{i=1}{\sum }}{\left(x_{i}-\bar{x}\right)}^{2}}$$

## 3. Results and Discussion

### 3.1. Uncoated Samples

Four sets, each consisting of four samples, were produced by the mechanical finishing methods: polishing, abrading with fleece, sandpapering and sandblasting. The fifth set consisted of four as-received substrates that were untreated and served as reference samples.

**Figure 4:**
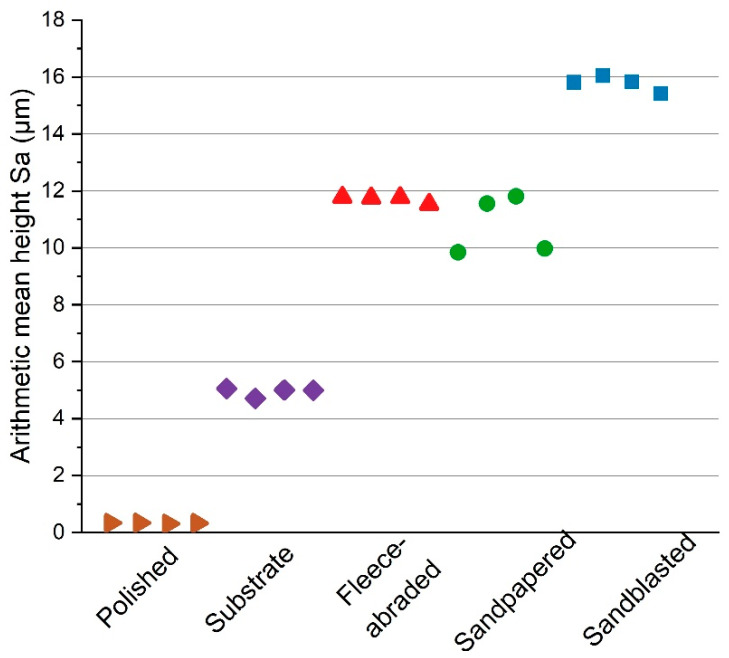
The experimentally determined arithmetic mean height (Sa) for each surface treatment method.

All samples prepared with the same method had the similar surface roughness parameter, namely, the arithmetic mean height Sa (Figure 4). The chosen surface finishing methods resulted in a broad range of values for Sa (between 0 and 16 µm). One sample out of four per each method was used for the further characterisation of the uncoated surfaces.

**Figure 5:**
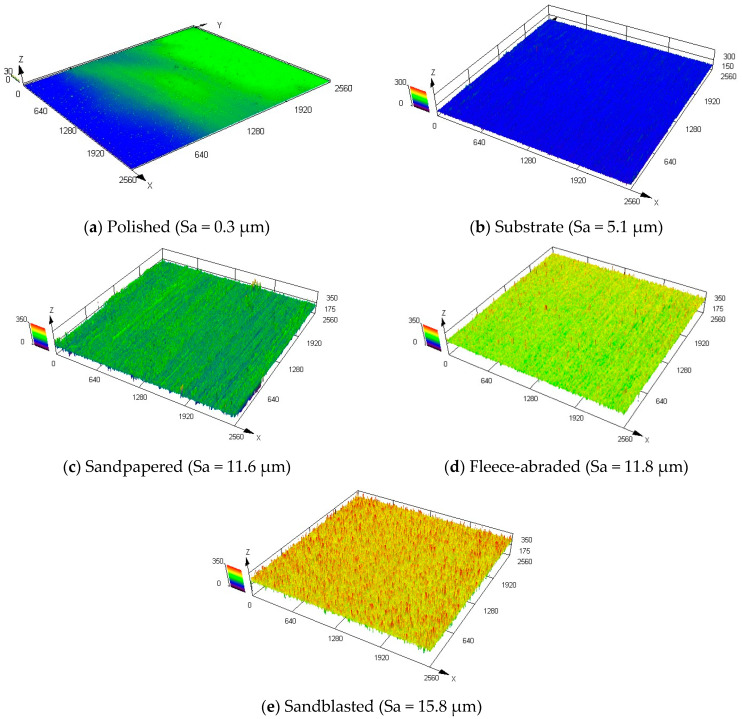
Morphologies of the uncoated samples.

A qualitative overview of the morphology of the examined surfaces, done via confocal laser scanning microscope, for the middle measurement points of the samples, is shown in Figure 5. The samples were arranged according to the growing average Sa value. The untreated substrate and the polished sample (Figure 5a,b) had a uniform profile with relatively low roughness, whereas the fleece-abraded and sandblasted samples (Figure 5d,e) were much rougher. The sandpapered sample had a rough surface that was also strongly directional-oriented (Figure 5c).

The selected roughness parameters, measured for each uncoated sample, are summarized in Table 2. As mentioned previously, the initial state of the surface before the treatment corresponds to the substrate. Remarkable for the substrate was that the maximum peak height (Sp) was about four times higher than the maximum valley depth (Sv). These, together with the high mean width of the profile elements (RSm = 100 μm), were produced due to the mechanical rolling of the substrate metal sheet. In general, the surface structure of the substrate was quite uniform (Figure 5b).

Polishing the surface resulted in the lowest areal roughness parameters among the considered samples, while the arithmetic mean height approached almost zero (Sa = 0.3 μm). As expected, the surface of the polished sample was smooth, with the lowest maximum height (Sz = 75.3 μm) and the broadest profile elements (RSm = 178.4 μm).

As the substrates were roughened, the arithmetic mean height of the samples increased. Thus, using the sandpaper led to an Sa value of 11.6 μm. The sandpapered sample had large standard deviations of Sa and Sq, indicating the significant differences in the height profile. These were reinforced by the much higher peaks, since the Sp was three times higher than the Sv, characterising the structure of the sandpapered sample as strongly non-uniform and directionally oriented.

Using the abrasive fleece resulted in the similar arithmetic mean height (Sa = 11.8 μm) as in the previous case with the sandpaper. The major difference was that the directional orientation of the surface treatment method was less pronounced in case of the fleece-abraded sample. The maximum peak height (Sp) was of the same order of magnitude as the maximum valley depth (Sv), which combined with the lowest mean width of the profile elements (RSm = 73.8 μm) spoke for the uniformly roughened surface structure.

The sandblasted sample, with the largest arithmetic mean height (Sa = 15.8 μm) and the largest maximum height (Sz = 479.7 μm), exhibited the roughest surface of all the tested treatment methods. However, sandblasting created a uniformly rough surface.

The wettability of the uncoated samples was characterised by measuring the apparent contact angles of water. The measurement results are shown in Table 3. The polished sample displayed the lowest contact angle of water $\theta_{ap,w}$ = 78° and, together with the substrate and fleece-abraded samples, were classified as hydrophilic surfaces. This reduction of the contact angle by polishing the sample does not correspond to the results published by Torrisi and Scolaro [24], who reported a slight contact angle increase, from 95° to 99°, by polishing the sample from 0.1 µm to 0.028 µm. However, these authors applied a chemical–mechanical polishing solution, used for polishing optical components in order to evenly remove the oxide layer from the surface. Thus, a very fine surface structure was created. The reduction of the contact angle in the present study could be caused by the newly air-formed oxide layer which distorted the surface profile.

Hydrophobic surfaces were achieved by sandblasting and sandpapering, with the latter exhibiting the highest contact angle $\theta_{ap,w}$ = 106°. It is worthwhile noticing that, although the sandpapered and fleece-abraded samples had the similar roughness parameters (Sa = 11.6 μm and Sa = 11.8 μm, respectively), their contact angles were considerably different. This difference is attributed to the strong anisotropy of the surface texture of the sandpapered sample, which is also visible from Figure 5c. These results emphasize that not only roughness, but also the surface treatment method has a significant impact on the wettability. 

Presented in Table 3, the ratio between the orthogonally measured base diameters of the third and the eighth droplets (BD3/BD8), both placed in the centres of each sample, helped to estimate whether the sample surface had an isotropic or anisotropic wettability. In the case of isotropic wetting behaviour, the base diameters measured from two orthogonal directions are similar. In its turn, for anisotropic wettability, the droplet base diameter measured in parallel to the surface texture direction (here, BD8) is different from the one measured perpendicular to the surface texture direction (here, BD3) [32,33]. 

When the ratio BD3/BD8 was close to one and was, in our case, in the range of 0.85–1.15, then the wettability was isotropic, as it was in the case with the polished sample and substrate. Due to the directionally oriented production of the metal sheet by mechanical rolling, the surface texture of the substrate was anisotropic. However, the texture anisotropy was not detected in wettability measurements. At a ratio of BD3/BD8 much higher or much lower than one and out of the above-specified range, the wettability was considered anisotropic, as by the sandpapered and fleece-abraded samples. Their surface texture was oriented in one direction (visible in Figure 5c,d) and was also anisotropic. 

**Figure 6:**
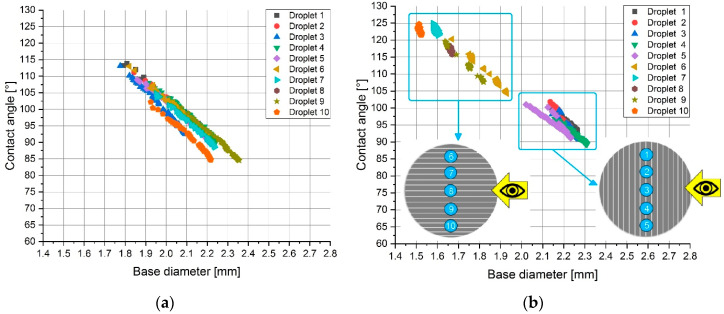
Dependency of the measured water contact angle on the base diameter for the (**a**) sandblasted (Sa = 15.8 μm) and (**b**) sandpapered samples (Sa = 11.6 μm).

Similar results, presented in Table 4, can be observed from the measured water contact angles perpendicular ($\theta_{ap,w\_1-5}$) and parallel ($\theta_{ap,w\_6-10}$) to the surface texture direction. These angles were considerably different ($\Delta \theta_{ap,w}$ = (10°–23°)) from each other in the case of the fleece-abraded and sandpapered samples, which indicated the strong anisotropy of the samples. Although the sandblasted sample had a ratio of BD3/BD8 much lower than one, the difference in the measured contact angles was minor. Apart from that, there was no grouping of the droplets in the dependence of the measuring direction as shown in Figure 6a. This let us conclude that the sandblasted sample was rather isotropic.

Following the change in the measured contact angles and droplet base diameters (Figure 6), within the selected timeframe of 45 s at each of the ten different droplet positions for the sandblasted and the sandpapered samples, a clear difference between the isotropic and anisotropic samples could be noticed. In the case of the isotropic sandblasted surface (Figure 6a), no clear difference between the sets of droplets 1–5 (measured perpendicular to the surface texture direction) and 6–10 (measured parallel to the surface texture direction) was observed. Typical for the anisotropic samples was the clear grouping of the droplets as shown for the sandpapered sample in Figure 6b. The explanation for this phenomenon is related to the fact that the directionally oriented surface treatment methods formed grooves with large maximum peak heights (e.g., Sp = 334.5 μm for the sandpapered sample).

**Figure 7:**
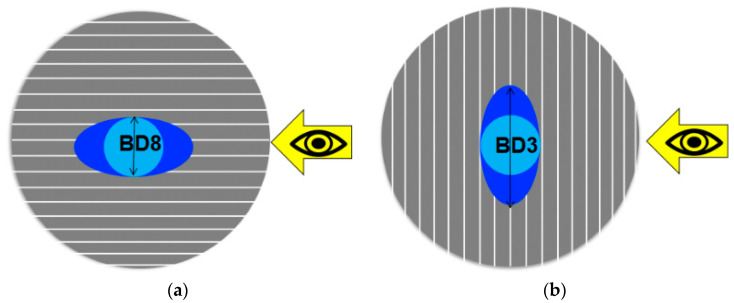
The schematic of the water droplet spreading in case of the measuring direction (**a**) parallel and (**b**) perpendicular to the surface texture.

The grooves served as barriers which prevented water droplets from spreading freely across the grooves, as shown schematically in Figure 7 for the case of droplet 8 (BD8). For droplet 8, the measured base diameter remained quite unchanged, but the contact angle of the drop increased [33]. While measuring the water contact angle perpendicular to the surface structure (BD3), the contact angle decreased and the base diameter increased due to the capillary force which made the water droplet spread along the groove.

**Figure 8:**
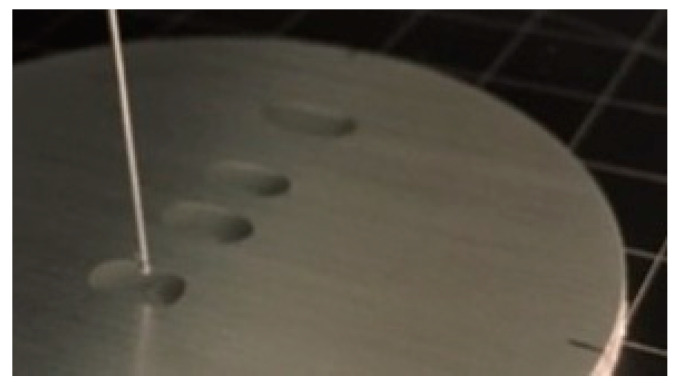
Oil droplets on the substrate.

Despite the different roughness and structures, the contact angles of oil could not be measured on any of the uncoated samples as exemplified in Figure 8. Due to the lower surface tension of oil compared to water, the capillary force accelerated the propagation of the oil droplet along the groove. Therefore, the oil penetrated into the surface structures and completely wetted the surface.

### 3.2. Coated Samples

Coating the prepared samples with the amphiphobic layer helped to produce significantly rougher surfaces with the arithmetic mean heights in the range of Sa = 13.4–29 μm. The maximum Sa value achieved by the mechanical treatment of the uncoated sample was 15.8 µm, whereas the coated sample had almost twice as high a value of Sa = 29 µm.

**Figure 9:**
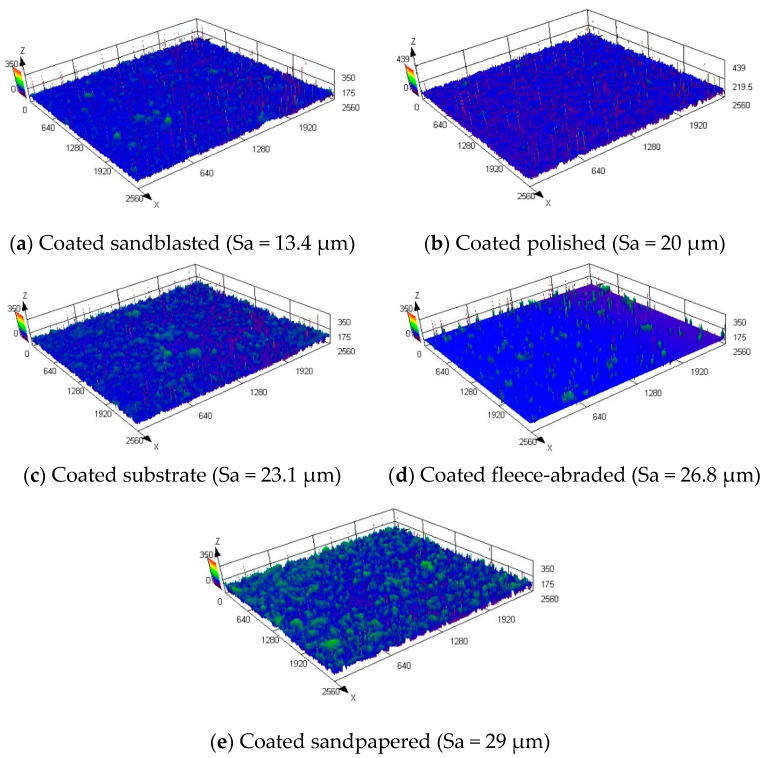
Morphologies of the coated samples.

The digital reproductions of the morphologies of the coated samples, made via microscope, are shown in Figure 9. Mainly the random agglomeration of the coating particles contributed to the roughening, since the initial surface under the coating was not observed and the previous order of the uncoated samples, sorted by the increasing Sa value (as in Figure 5), was no more kept.

In addition to the figures of the samples’ surfaces, the quantitative description by the selected areal roughness parameters are summarized in Table 5. The determination of the individual profile elements in the horizontal direction was not possible; therefore, the mean width of the profile elements (RSm) was excluded from the analysis.

The coated sample, which had a base that was a sandblasted surface, had the lowest values of the arithmetic mean height (Sa = 13.4 μm), root mean squared height (Sq = 18.6 μm) and maximum peak height (Sp = 222.6 μm). This was remarkable, since the uncoated sandblasted sample exposed the roughest surface. During the coating, the particles within the coating fluid had filled the micro holes that were created arbitrarily by the sandblasting, forming a smooth, uniform surface.

In general, the originally anisotropic samples (fleece-abraded and sandpapered) formed significantly rougher surfaces after being coated than the originally isotropic samples (sandblasted and polished). 

The wetting behaviour of the coated samples was again characterised through the measurements of the apparent contact angles of the two tested liquids: demineralized water ($\theta_{ap,w\_coated}$) and rapeseed oil ($\theta_{ap,o\_coated}$), presented in Table 6.

With the contact angles of water higher than 150° and the contact angles of oil in the range between 139° and 150° for all samples, the coating resulted in the creation of the superhydrophobic and oleophobic surfaces according to the criteria mentioned in the Section 1. The mechanical surface treatment methods, conducted before the coating, appeared to play a nonessential role, since all the coated samples exhibited similar contact angles of water $\theta_{ap,w\_coated}$ = 162°–173° as well as of oil, $\theta_{ap,o\_coated}$ = 139°–150°, in a similar range of standard deviations.

**Figure 10:**
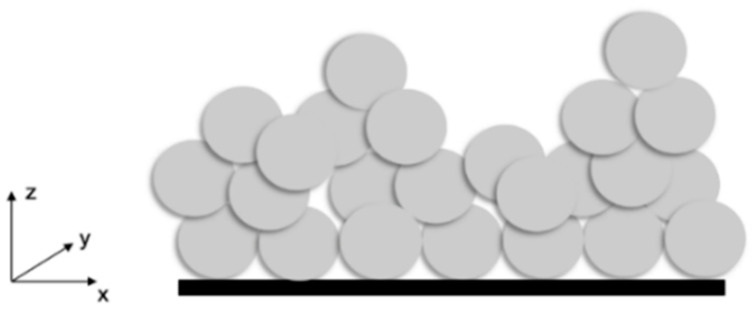
Schematic of the coating particles’ arrangement.

Compared to the water contact angles of the uncoated samples, the contact angles almost doubled after the application of the coating. Multiscale roughness was created due to the agglomeration of the coating particles (Figure 10) which favoured the high contact angles [34,35]. Thus, a porous structure with a round, hilly surface was formed as shown in Figure 9e. The inclusion of air is probable, indicating a Cassie–Baxter wetting state.

**Figure 11:**
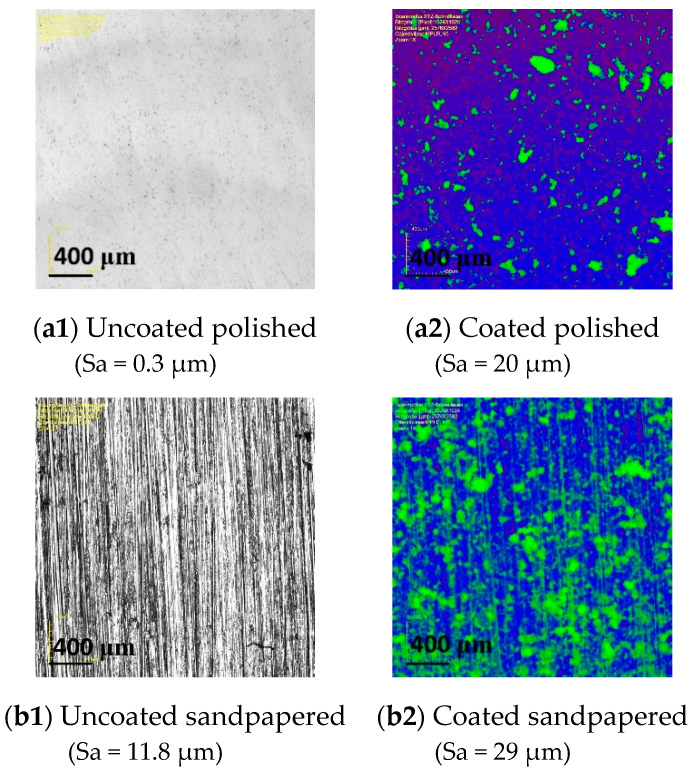
Microscope images of the uncoated (black–white) and coated (false colour) polished (**a1**,**a2**) and sandpapered (**b1**,**b2**) samples.

Microscopic images of the polished and sandpapered samples illustrate the coating particles’ arrangement in comparison to the initial uncoated surfaces (Figure 11). The particles agglomerated on the surface arbitrarily and independently of the initial surface structure. Even in the case of the originally strongly anisotropic sandpapered sample (Figure 11b1), its base structure was completely cancelled by the coating (Figure 11b2).

All the coated samples had an isotropic wetting behaviour. The oil contact angles of the coated samples, measured perpendicular and parallel to the surface texture direction (Table 7), showed slightly differing values between $\theta_{ap,o\_coated\_1-5}$ and $\theta_{ap,o\_coated\_6-10}$. Nevertheless, these differences are attributed rather to the local surface energy and local surface structure.

The temperature stability of the coating was tested by heating up the coated substrate three times to the temperature of 50 °C for 1 h. After each heating step, the water contact angles were measured, and the results are presented in Table 8. Under the influence of the increased temperature, the contact angles decreased from 175° to 138°, thus transferring the coated surface from the superhydrophobic to the hydrophobic region. 

The main drop in the contact angle (around 30°) was measured after the first heating cycle, while afterwards the contact angle remained almost constant. A similar behaviour was observed with another coated substrate, heated up for only 10 min at 50 °C (Table 9). The water contact angle decreased by 26° within just ten minutes of heating. On the other side, the elevated temperature did not affect the oil contact angles, which stayed unchanged (about 135°) during the testing. Therefore, heating the coated substrate at 50 °C may remove from the coating the components, that are water-repellent but have no influence on oil/surface interaction.

**Figure 12:**
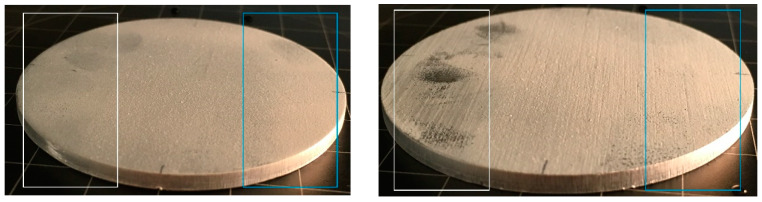
Visual comparison of the coated samples after the removal of the stronger (marked with white) and weaker (marked with blue) adhesive tapes.

The coating adhesion was initially tested by the tape test using a stronger (to the left side of the coated samples) and weaker (to the right side of the coated samples) adhesive tape. The middle part of the samples was intentionally left unchanged for comparison (Figure 12).

After using the stronger adhesive tape (white), a significant removal of the coating was observed for all coated samples. Better adhesion of the coating towards the originally rough samples (fleece-abraded and sandblasted), which was expected, could not be observed. A strong adhesion of the tape to the left side of the coated fleece-abraded sample (Figure 12b) was the most noticeable of all the coated samples. Nevertheless, the measured water contact angles, even in this extreme case, were quite similar ($\theta_{ap,w\_white}$ = 153° and $\theta_{ap,w\_blue}$ = 150°) as presented in Table 10. 

The coated as-received substrate showed significant decrease of the contact angle for both tape-tested parts of the surface, and, in addition, high standard deviations were observed for the values. The other tested samples exhibited contact angles of around 150° for the original surface as well as for the tape-tested surface, indicating that the coating layer was sufficiently thick to render the surface superhydrophobic, in spite of the partial removal of the coating. The significantly different behaviour of the coated as-received substrate may indicate that mechanical surface pre-treatments are beneficial for the coating adhesion. This preliminary adhesion test indicated the need for a more detailed investigation of the abrasive wear of the coating, especially in regard to the anisotropic structures, which is a partial outlook of the present study.

## 4. Conclusions and Outlook

In the first part of this experimental study, five differently mechanically treated aluminium surfaces (as-received substrate, polished, sandpapered, fleece-abraded and sandblasted samples) were characterised regarding their roughness and static wettability. Special attention was given to the correlation between the applied surface finishing method and isotropy of the samples’ surface. 

In the second part of this work, an amphiphobic coating, based on a fluoroacrylic solution with fluorinated nanoparticles, was sprayed on the samples in order to achieve the water and oil repellence. The influence of roughness and surface preparation methods on the wettability of both uncoated and coated samples was analysed by measuring the apparent contact angles of demineralized water and rapeseed oil.

The applied mechanical surface treatment methods led to a roughness in the range of Sa = 0.3 µm–15.8 µm and to the water contact angles of $\theta_{ap,w}$ = 78°–106°. By coating the samples, the arithmetic mean height increased more than twice to Sa = 13.3 µm–29 µm. The results indicated that the coating completely cancelled the surface properties related to the previous mechanical treatment (e.g., structure, anisotropy, wettability). Furthermore, the originally anisotropic samples (i.e., fleece-abraded and sandpapered) formed significantly rougher surfaces after being coated than the originally isotropic samples (i.e., sandblasted, polished).

The applied coating increased the water contact angles to $\theta_{ap,w\_coated}$ = 162°–173°, classifying all the coated samples as superhydrophobic according to this criteria. Even more pronounced effects were measured with the contact angles of oil which changed from extremely low for the uncoated samples to the values of $\theta_{ap,o\_coated}$ = 139°–150°, making all the coated samples oleophobic. 

The temperature stability tests indicated that exposure of the coated substrate to an elevated temperature of 50 °C decreased the water contact angles by approximately 26° within the first few minutes of heating. However, extended heating time or cyclic tests of heating and cooling did not lead to the further water contact angle decrease. On the other hand, the elevated temperature did not affect the oil contact angles, which stayed unchanged (about 135°) during the testing. 

Finally, preliminary adhesive tests showed that, although part of the coating could be easily removed with the adhesive tape, the water contact angles remained quite high (approximately 150°), which signified a sufficient thickness of the coating layer and a promising mechanical stability. For a more detailed characterization of the coating layer stability, further material tests are required. 

## Figures and Tables

**Table 1:** The selected roughness parameters for the characterization of the prepared samples [27,28].

Roughness Parameter	Equation
Arithmetic mean height (Sa)	(1) $Sa=\frac{1}{A}\overset{\;}{\underset{A}{\iint }}\left|Z\left(x,y\right)\right|dxdy$
Root mean squared height (Sq)	(2) $Sq=\sqrt{\frac{1}{A}\overset{\;}{\underset{A}{\iint }}Z^{2}\left(x,y\right)dxdy}$
Maximum height (Sz)	(3) Sz=Sp+Sv
Maximum peak height (Sp)	(4) Sp=max(Z(x,y))
Maximum valley depth (Sv)	(5) Sv=min(Z(x,y))
Mean width of the profile elements (RSm)	(6) $RSm=\frac{1}{m}\sum_{i=1}^{m}X_{si}$

**Table 2:** Overview of the averaged areal and linear roughness parameters of the uncoated samples, with the indicated standard deviations (σ).

Uncoated Samples	Polished (Sa = 0.3 μm)		Substrate (Sa = 5.1 μm)		Sandpapered (Sa = 11.6 μm)		Fleece-Abraded (Sa = 11.8 μm)		Sandblasted (Sa = 15.8 μm)	
	Average	±σ	Average	±σ	Average	±σ	Average	±σ	Average	±σ
Sa (μm)	0.3	0.1	5.1	0.1	11.6	1.0	11.8	0.3	15.8	0.2
Sq (μm)	0.7	0.2	6.9	0.1	16.7	2.2	15.3	0.4	22.9	0.4
Sz (μm)	75.3	26.0	367.1	55.1	445.8	6.0	457.2	6.1	479.7	8.5
Sp (μm)	62.1	17.4	288.4	57.2	334.5	35.0	239.2	51.9	245.2	17.7
Sv (μm)	13.2	16.3	78.6	11.3	111.3	37.2	218.1	53.6	234.5	12.5
RSm(μm)	178.4	38.8	100.0	5.7	92.2	15.3	73.8	4.3	74.9	4.1

**Table 3:** The apparent contact angles of water ($\theta_{ap,w}$) and the ratios between the base diameters of the third to the eighth droplets (BD3/BD8) with the indicated standard deviations (σ) for the uncoated samples.

Uncoated Samples	$\theta_{ap,w}$ (°)		BD3/BD8 (-)	
	Average	±σ	Average	±σ
Polished (Sa = 0.3 μm)	78.0	6	0.88	0.22
Substrate (Sa = 5.1 μm)	86.2	2	1.06	0.09
Sandpapered (Sa = 11.6 μm)	105.5	13	1.35	0.42
Fleece-abraded (Sa = 11.8 μm)	81.9	7	1.16	0.23
Sandblasted (Sa = 15.8 μm)	94.3	3	0.69	0.65

**Table 4:** The apparent contact angles of water measured perpendicular ($\theta_{ap,w\_1-5}$) and parallel ($\theta_{ap,w\_6-10}$) to the surface texture direction for the uncoated samples.

Uncoated Samples	Polished (Sa = 0.3 μm)		Substrate (Sa = 5.1 μm)		Sandpapered (Sa = 11.6 μm)		Fleece-Abraded (Sa = 11.8 μm)		Sandblasted (Sa = 15.8 μm)	
	Average	±σ	Average	±σ	Average	±σ	Average	±σ	Average	±σ
$\theta_{ap,w\_1-5}\;$ (°)	79.8	5.3	84.9	1.6	94.0	2.1	76.6	2.1	96.3	1.2
$\theta_{ap,w\_6-10}\;$ (°)	76.1	7.0	87.5	2.0	116.9	6.1	87.2	5.5	92.2	3.7

**Table 5:** Overview of the averaged areal roughness parameters of the coated samples with the indicated standard deviations (σ).

Coated Samples	Coated Sandblasted (Sa = 13.4 μm)		Coated Polished (Sa = 20 μm)		Coated Substrate (Sa = 23.1 μm)		Coated Fleece-Abraded (Sa = 26.8 μm)		Coated Sandpapered (Sa = 29 μm)	
	Average	±σ	Average	±σ	Average	±σ	Average	±σ	Average	±σ
Sa (μm)	13.4	1.0	20.0	5.1	23.1	1.9	26.8	0.7	29.0	1.2
Sq (μm)	18.6	1.1	33.1	5.4	28.5	1.9	33.5	0.8	35.4	1.4
Sz (μm)	406.7	4.9	413.4	12.4	403.3	4.7	395.9	8.9	408.0	8.5
Sp (μm)	222.6	9.1	336.1	8.9	273.7	3.1	274.9	8.5	284.1	8.0
Sv (μm)	184.2	8.6	77.3	4.9	129.6	2.5	120.9	7.2	123.8	12.2

**Table 6:** The apparent contact angles of water ($\theta_{ap,w\_coated}$) and of oil ($\theta_{ap,o\_coated}$) with the indicated standard deviations (σ) for the coated samples.

Coated Samples	$\theta_{ap,w\_coated}$ (°)		$\theta_{ap,o\_coated}$ (°)	
	Average	±σ	Average	±σ
Coated sandblasted (Sa = 13.4 μm)	170.0	16.5	146.0	3.8
Coated polished (Sa = 20 μm)	161.7	20.0	149.7	5.5
Coated substrate (Sa = 23.1 μm)	172.0	16.0	146.0	3.8
Coated fleece-abraded (Sa = 26.8 μm)	173.3	14.4	145.0	2.4
Coated sandpapered (Sa = 29 μm)	169.6	14.4	139.3	11.3

**Table 7:** The apparent contact angles of oil, measured perpendicular ($\theta_{ap,o\_coated\_1-5}$) and parallel ($\theta_{ap,o\_coated\_6-10}$) to the surface texture direction, for the coated samples.

Coated Samples	Coated Sandblasted (Sa = 13.4 μm)		Coated Polished (Sa = 20 μm)		Coated Substrate (Sa = 23.1 μm)		Coated Fleece-Abraded (Sa = 26.8 μm)		Coated Sandpapered (Sa = 29 μm)	
	Average	±σ	Average	±σ	Average	±σ	Average	±σ	Average	±σ
$\theta_{ap,o\_coated\_1-5}\;$(°)	149.7	2.0	154.4	2.9	149.3	2.0	144.0	2.5	149.7	1.2
$\theta_{ap,o\_coated\_6-10}\;$(°)	142.7	0.9	145.1	2.4	142.7	0.9	146.1	2.1	128.9	4.2

**Table 8:** The measured water contact angles for the coated substrate at room temperature and after the temperature tests.

Coated Sample	Steps	Temperature, Time	$\theta_{ap,w\_coated}$ (°)	
			Average	±σ
Coated Substrate (Sa = 15.7 μm)	1	20 °C	175.2	9.6
	2	50 °C, 1 h	143.5	6.2
	3	50 °C, 1 h	139.0	14.9
	4	50 °C, 1 h	138.4	5.4

**Table 9:** Comparison of the contact angles of water ($\theta_{ap,w\_coated}$) and of oil ($\theta_{ap,o\_coated}$) at room temperature and after the temperature test at 50 °C for 10 min.

Coated Sample	Steps	Temperature, Time	$\theta_{ap,w\_coated}$ (°)		$\theta_{ap,o\_coated}$ (°)	
			Average	±σ	Average	±σ
Coated Substrate (Sa = 14.1 μm)	1	20 °C	175.7	7.1	135.4	14.4
	2	50 °C, 10 min	150.2	16.6	134.3	3.2

**Table 10:** Comparison of the water contact angles after the tape test on the parts, where the stronger ($\theta_{ap,w\_white}$) and weaker adhesive tapes ($\theta_{ap,w\_blue}$) were applied as well as on the untouched coated part ($\theta_{ap,w\_coated}$) for the tested samples.

Coated Samples	Coated Sandblasted (Sa = 10.6 μm)		Coated Substrate (Sa = 15.4 μm)		Coated Fleece-Abraded (Sa = 26.8 μm)	
	Average	±σ	Average	±σ	Average	±σ
$\theta_{ap,w\_white}$ (°)	152.6	7.5	131.5	48.3	153.4	4.8
$\theta_{ap,w\_coated}$ (°)	155.1	7.1	153.0	15.7	163.5	15.1
$\theta_{ap,w\_blue}$ (°)	145.4	0.7	109.3	56.4	149.5	5.6
